# Supplementary material for: Differential expression of gluconeogenesis-related transcripts in a freshwater zooplankton model organism suggests a role of the Cori cycle in hypoxia tolerance
Source: PLoS One. 2023 Aug 8;18(8):e0284679. doi: 10.1371/journal.pone.0284679 (PMC10409257; doi:10.1371/journal.pone.0284679)
Supplement: Peer review history

# Peer review: Decision Letter 0

**DOI:** 10.1371/journal.pone.0284679.r001

**Authors:** Bashir Sajo Mienda

27 Apr 2023

PONE-D-23-10222Differential Expression of Gluconeogenesis-Related Transcripts in a Freshwater Zooplankton Model Organism Suggests a Role of the Cori Cycle in Hypoxia TolerancePLOS ONE

Dear Dr. Yampolsky,

Thank you for submitting your manuscript to PLOS ONE. After careful consideration, we feel that it has merit but does not fully meet PLOS ONE’s publication criteria as it currently stands. Therefore, we invite you to submit a revised version of the manuscript that addresses the points raised during the review process.

Please submit your revised manuscript by Jun 11 2023 11:59PM. If you will need more time than this to complete your revisions, please reply to this message or contact the journal office at plosone@plos.org. When you're ready to submit your revision, log on to https://www.editorialmanager.com/pone/ and select the 'Submissions Needing Revision' folder to locate your manuscript file.

Please include the following items when submitting your revised manuscript:

- A rebuttal letter that responds to each point raised by the academic editor and reviewer(s). You should upload this letter as a separate file labeled 'Response to Reviewers'.
- A marked-up copy of your manuscript that highlights changes made to the original version. You should upload this as a separate file labeled 'Revised Manuscript with Track Changes'.
- An unmarked version of your revised paper without tracked changes. You should upload this as a separate file labeled 'Manuscript'.

If you would like to make changes to your financial disclosure, please include your updated statement in your cover letter. Guidelines for resubmitting your figure files are available below the reviewer comments at the end of this letter.

If applicable, we recommend that you deposit your laboratory protocols in protocols.io to enhance the reproducibility of your results. Protocols.io assigns your protocol its own identifier (DOI) so that it can be cited independently in the future. For instructions see: https://journals.plos.org/plosone/s/submission-guidelines#loc-laboratory-protocols. Additionally, PLOS ONE offers an option for publishing peer-reviewed Lab Protocol articles, which describe protocols hosted on protocols.io. Read more information on sharing protocols at https://plos.org/protocols?utm_medium=editorial-email&utm_source=authorletters&utm_campaign=protocols.

We look forward to receiving your revised manuscript.

Kind regards,

Bashir Sajo Mienda, PhD

Academic Editor

PLOS ONE

Journal Requirements:

When submitting your revision, we need you to address these additional requirements.

1. Please ensure that your manuscript meets PLOS ONE's style requirements, including those for file naming. The PLOS ONE style templates can be found at 

https://journals.plos.org/plosone/s/file?id=wjVg/PLOSOne_formatting_sample_main_body.pdf and 

https://journals.plos.org/plosone/s/file?id=ba62/PLOSOne_formatting_sample_title_authors_affiliations.pdf

2. We note that the grant information you provided in the ‘Funding Information’ and ‘Financial Disclosure’ sections do not match. 

When you resubmit, please ensure that you provide the correct grant numbers for the awards you received for your study in the ‘Funding Information’ section.

3. Please amend either the abstract on the online submission form (via Edit Submission) or the abstract in the manuscript so that they are identical.

4. We note that Figure 1 in your submission contain copyrighted image. All PLOS content is published under the Creative Commons Attribution License (CC BY 4.0), which means that the manuscript, images, and Supporting Information files will be freely available online, and any third party is permitted to access, download, copy, distribute, and use these materials in any way, even commercially, with proper attribution. For more information, see our copyright guidelines: http://journals.plos.org/plosone/s/licenses-and-copyright.

We require you to either (1) present written permission from the copyright holder to publish these figures specifically under the CC BY 4.0 license, or (2) remove the figures from your submission:

a. You may seek permission from the original copyright holder of Figure 1 to publish the content specifically under the CC BY 4.0 license. 

We recommend that you contact the original copyright holder with the Content Permission Form (http://journals.plos.org/plosone/s/file?id=7c09/content-permission-form.pdf) and the following text:

“I request permission for the open-access journal PLOS ONE to publish XXX under the Creative Commons Attribution License (CCAL) CC BY 4.0 (http://creativecommons.org/licenses/by/4.0/). Please be aware that this license allows unrestricted use and distribution, even commercially, by third parties. Please reply and provide explicit written permission to publish XXX under a CC BY license and complete the attached form.”

Please upload the completed Content Permission Form or other proof of granted permissions as an ""Other"" file with your submission. 

In the figure caption of the copyrighted figure, please include the following text: “Reprinted from [ref] under a CC BY license, with permission from [name of publisher], original copyright [original copyright year].”

b. If you are unable to obtain permission from the original copyright holder to publish these figures under the CC BY 4.0 license or if the copyright holder’s requirements are incompatible with the CC BY 4.0 license, please either i) remove the figure or ii) supply a replacement figure that complies with the CC BY 4.0 license. Please check copyright information on all replacement figures and update the figure caption with source information. If applicable, please specify in the figure caption text when a figure is similar but not identical to the original image and is therefore for illustrative purposes only.

5. Please review your reference list to ensure that it is complete and correct. If you have cited papers that have been retracted, please include the rationale for doing so in the manuscript text, or remove these references and replace them with relevant current references. Any changes to the reference list should be mentioned in the rebuttal letter that accompanies your revised manuscript. If you need to cite a retracted article, indicate the article’s retracted status in the References list and also include a citation and full reference for the retraction notice.

[Note: HTML markup is below. Please do not edit.]

Reviewers' comments:

Reviewer's Responses to Questions

**Comments to the Author**

1. Is the manuscript technically sound, and do the data support the conclusions?

The manuscript must describe a technically sound piece of scientific research with data that supports the conclusions. Experiments must have been conducted rigorously, with appropriate controls, replication, and sample sizes. The conclusions must be drawn appropriately based on the data presented. 

Reviewer #1: Yes

**********

2. Has the statistical analysis been performed appropriately and rigorously? 

Reviewer #1: Yes

**********

3. Have the authors made all data underlying the findings in their manuscript fully available?

The PLOS Data policy requires authors to make all data underlying the findings described in their manuscript fully available without restriction, with rare exception (please refer to the Data Availability Statement in the manuscript PDF file). The data should be provided as part of the manuscript or its supporting information, or deposited to a public repository. For example, in addition to summary statistics, the data points behind means, medians and variance measures should be available. If there are restrictions on publicly sharing data—e.g. participant privacy or use of data from a third party—those must be specified.

Reviewer #1: Yes

**********

4. Is the manuscript presented in an intelligible fashion and written in standard English?

PLOS ONE does not copyedit accepted manuscripts, so the language in submitted articles must be clear, correct, and unambiguous. Any typographical or grammatical errors should be corrected at revision, so please note any specific errors here.

Reviewer #1: Yes

**********

5. Review Comments to the Author

Please use the space provided to explain your answers to the questions above. You may also include additional comments for the author, including concerns about dual publication, research ethics, or publication ethics. (Please upload your review as an attachment if it exceeds 20,000 characters)

Reviewer #1: Review of Yampolsky et al.

This manuscript describs a gene expression test for differential and tissue-specific expression of gluconeogenesis-specific genes during conditions of low oxygen (hypoxia), as compared to normal oxygen levels. The study shows that some effects are visible, but overall does not provide strong results of differential expression in heads and body. Only fructose-1,6-bisphosphatase showed differential expression. The authors conclude that their research is consistent with the hypothesis that Daphnia is able to tolerate hypoxia through the Cori cycle. The idea being that the body tissue generates glucose and NAD+ which is used in the brain and locomotory muscles in the head. Therefore ATP production is enabled.

The study explains the reasoning and logic of the design very well. The text is clearly written and easy to follow. I have only minor points to comment.

1. It is not clear why head and body was used as the two tissues being studied. Also, looking at a picture of daphnia, it seems hard to draw a clear line between head and body. I would appreciate a bit more details on both points.

2. The authors suggest that the adipose tissue may play a role in the body for GNG. What is the reasoning for this? Are there examples in the literature.

3. The authors have a very black and white picture of their quantitative data. Statements like: "did not differ among clone", "there is no interaction effects", "no evidence for differential expression", ...) are not justified. If a test is not significant it does not mean that there is no difference. In modern scientific writing, such statements should be avoided. Stating that a difference is not significant is ok to say.

4. what are the numbers in the graph of Fig. 2.

5. It would be nice to be more clear about sample sizes. E.g. in the statistical tables and figures.

6. The conclusion is more a summary than a conclusion. but it is a good summary. Maybe place it as a summary at the right place and think about a more interesting conclusion. E.g. think about what this means for other plankton dwellers or more generally invertebrates.

**********

6. PLOS authors have the option to publish the peer review history of their article (what does this mean?). If published, this will include your full peer review and any attached files.

If you choose “no”, your identity will remain anonymous but your review may still be made public.

**Do you want your identity to be public for this peer review?** For information about this choice, including consent withdrawal, please see our Privacy Policy.

Reviewer #1: No

**********

[NOTE: If reviewer comments were submitted as an attachment file, they will be attached to this email and accessible via the submission site. Please log into your account, locate the manuscript record, and check for the action link "View Attachments". If this link does not appear, there are no attachment files.]

While revising your submission, please upload your figure files to the Preflight Analysis and Conversion Engine (PACE) digital diagnostic tool, https://pacev2.apexcovantage.com/. PACE helps ensure that figures meet PLOS requirements. To use PACE, you must first register as a user. Registration is free. Then, login and navigate to the UPLOAD tab, where you will find detailed instructions on how to use the tool. If you encounter any issues or have any questions when using PACE, please email PLOS at figures@plos.org. Please note that Supporting Information files do not need this step.

# Author comment: Author response to Decision Letter 0

**DOI:** 10.1371/journal.pone.0284679.r002

19 Jul 2023

Reviewer #1: Review of Yampolsky et al.

1. It is not clear why head and body was used as the two tissues being studied. Also, looking at a picture of daphnia, it seems hard to draw a clear line between head and body. I would appreciate a bit more details on both points.

 We added reasoning why we believe the recipient tissues are located in the head and donor tissues in the body of Daphnia. We also added images (Fig 1B,C) to illustrate this.

2. The authors suggest that the adipose tissue may play a role in the body for GNG. What is the reasoning for this? Are there examples in the literature.

We references explaining why it is possible to regard the arthropode fat body is a functional analog of mammalian liver and adipose tissue, specifically with respect to GNG.

3. The authors have a very black and white picture of their quantitative data. Statements like: "did not differ among clone", "there is no interaction effects", "no evidence for differential expression", ...) are not justified. If a test is not significant it does not mean that there is no difference. In modern scientific writing, such statements should be avoided. Stating that a difference is not significant is ok to say.

We are not sure we fully agree. Modern scientific writing offers ample warnings not to make positive statements about the existence of an effect even when a test is formally significant (e.g. due to small effect magnitude or low priors). On the other hand, it is, usually, safe to say that an effect does not exist (or there are no reasons to believe that it exists) when tests yield non-significant results. It is always conservative to assume no effect that to suspect one. We did change the language, though, from “no effect” to “no evidence of effect”.

4. what are the numbers in the graph of Fig. 2.

We will double-check the submission that the axes labels are not lost in pdf conversion – they are in place on our end. 

5. It would be nice to be more clear about sample sizes. E.g. in the statistical tables and figures.

We added sample sizes explicitly.

6. The conclusion is more a summary than a conclusion. but it is a good summary. Maybe place it as a summary at the right place and think about a more interesting conclusion. E.g. think about what this means for other plankton dwellers or more generally invertebrates.

 We added admittedly a somewhat speculative statement about more general significance.

# Editor report: Decision Letter 1

**DOI:** 10.1371/journal.pone.0284679.r003

**Authors:** Bashir Sajo Mienda

24 Jul 2023

Differential Expression of Gluconeogenesis-Related Transcripts in a Freshwater Zooplankton Model Organism Suggests a Role of the Cori Cycle in Hypoxia Tolerance

PONE-D-23-10222R1

Dear Dr. Yampolsky,

We’re pleased to inform you that your manuscript has been judged scientifically suitable for publication and will be formally accepted for publication once it meets all outstanding technical requirements.

Within one week, you’ll receive an e-mail detailing the required amendments. When these have been addressed, you’ll receive a formal acceptance letter and your manuscript will be scheduled for publication.

An invoice for payment will follow shortly after the formal acceptance. To ensure an efficient process, please log into Editorial Manager at http://www.editorialmanager.com/pone/, click the 'Update My Information' link at the top of the page, and double check that your user information is up-to-date. If you have any billing related questions, please contact our Author Billing department directly at authorbilling@plos.org.

If your institution or institutions have a press office, please notify them about your upcoming paper to help maximize its impact. If they’ll be preparing press materials, please inform our press team as soon as possible -- no later than 48 hours after receiving the formal acceptance. Your manuscript will remain under strict press embargo until 2 pm Eastern Time on the date of publication. For more information, please contact onepress@plos.org.

Kind regards,

Bashir Sajo Mienda, PhD

Academic Editor

PLOS ONE

Additional Editor Comments (optional):

Reviewers' comments:

# Editor report: Acceptance letter

**DOI:** 10.1371/journal.pone.0284679.r004

**Authors:** Bashir Sajo Mienda

28 Jul 2023

PONE-D-23-10222R1 

Differential Expression of Gluconeogenesis-Related Transcripts in a Freshwater Zooplankton Model Organism Suggests a Role of the Cori Cycle in Hypoxia Tolerance 

Dear Dr. Yampolsky:

I'm pleased to inform you that your manuscript has been deemed suitable for publication in PLOS ONE. Congratulations! Your manuscript is now with our production department. 

If your institution or institutions have a press office, please let them know about your upcoming paper now to help maximize its impact. If they'll be preparing press materials, please inform our press team within the next 48 hours. Your manuscript will remain under strict press embargo until 2 pm Eastern Time on the date of publication. For more information please contact onepress@plos.org.

If we can help with anything else, please email us at plosone@plos.org. 

Thank you for submitting your work to PLOS ONE and supporting open access. 

Kind regards, 

PLOS ONE Editorial Office Staff

on behalf of

Dr. Bashir Sajo Mienda 

Academic Editor

PLOS ONE
